# Supplementary material for: High-fat diet disturbs lipid raft/TGF-β signaling-mediated maintenance of hematopoietic stem cells in mouse bone marrow
Source: Nat Commun. 2019 Jan 31;10:523. doi: 10.1038/s41467-018-08228-0 (PMC6355776; doi:10.1038/s41467-018-08228-0)
Supplement: Supplementary file 1 — Supplementary Information [file 41467_2018_8228_MOESM1_ESM.pdf]

Supplementary Information

High-fat diet disturbs lipid raft/TGF-β signaling-mediated maintenance of hematopoietic stem cells in mouse bone marrow

Hermetet et al.

Supplementary Methods

Transplantations

For the SLAM transplantation study, mice were fed a CD or HFD for 4 weeks. SLAM cells (1,000 Ly.1 cells) were isolated by FACS and transplanted in competition with 200,000 BM cells from Ly.2 mice in lethally irradiated Ly.2 mice. PB and BM reconstitutions mediated by SLAM cells were analyzed 16 weeks after the transplantation. For the niche dependent reconstitution study, Ly.2 mice were fed CD or HFD for 4 weeks, and the animals were irradiated (900cGy) and transplanted with 200,000 Ly.1 total BM cells to analyze PB and BM reconstitutions, 16 weeks after the transplantation.

Immunofluorescence and microscopy

LSK-CD34$^-$ cells were stained with cell surface antibodies prior to cell sorting on glass slides and microscopy. For the study of several receptors on the cell surface, LR were stained with AF488-conjugated CTB (C34775, Thermo Fisher Scientific, 1 µg/mL) and we used biotin-conjugated c-Kit/CD117 (553353, BD Pharmingen, dilution ratio 1:50), IL3Rα (106003, Biolegend, dilution ratio 1:50), IL6Rα (115803, Biolegend, dilution ratio 1:50) antibodies and Streptavidin-AF555 (S32355, Thermo Fisher Scientific, dilution ratio 1:500). Images were acquired with an Axio Imager M2 (Zeiss) coupled with an Apotome.2 (×63 objective) and processed for colocalization studies (Fiji, NIH software).

Single-cell culture for cell division study

Sorted LSK-CD34$^-$ cells were deposited into 96-well U bottom plate at the rate of one cell per well, each containing 100µL Serum Free Expansion Medium (Stem Cell technology) supplemented with 10% fetal bovine serum (Dominique Dutscher), 100 U/mL penicillin, 100 µg/mL streptomycin, 250 ng/mL amphotericin B (PAN-Biotech), 20 ng/mL mouse stem cell factor, 50 ng/mL human thrombopoietin (Miltenyi Biotec) in the presence or absence of 10 ng/mL rTGF-β1 (7666-MB, R&D Biosystems). HSC were grown at 37°C (5% CO_2) and their survival and division monitored by microscopy.

Histochemistry

Hind limb bones were collected, stripped of soft tissue, fixed in 10% buffered formalin for 24 hours, processed and embedded in paraffin. Four-µm-thick sections were cut from paraffin-embedded samples and used for hematoxylin and eosin staining. After numeration using the Nanozoomer 2.0 HT and NDP scan software (Hamamatsu), the slides were processed; adipocytes were counted in the femoral head for each sample and their size evaluated.

ELISA

Mouse bones were crushed in a mortar with 750 µL of cold PBS. After centrifugation (10,000g, 10 min), cell supernatants were assayed in triplicate with ELISA for mouse TNF-α (88-7324), IL-1β (88-7013), CCL2 (88-7391), TGF-β1 (88-8350) and IL-10 (88-7104), according to the manufacturer's instructions (eBioscience).

Supplementary Figures

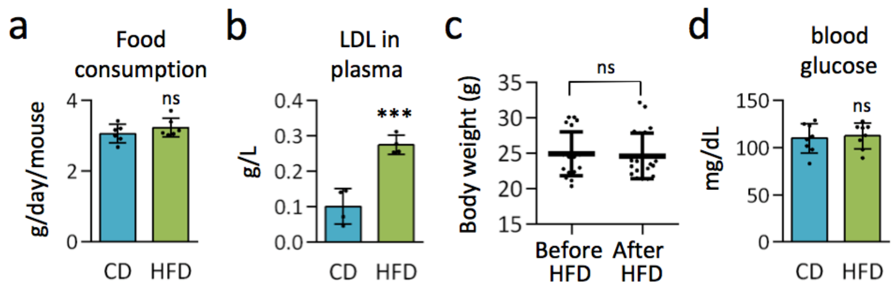

Supplementary Figure 1

A short-term HFD in mice increases LDL levels in PB, with no evidence of obesity or prediabetes. **a** Food consumption for CD and HFD mice. Total consumption is calculated during the 4 weeks of feeding and the mean is calculated in g/day/mouse, n=6 mice per diet group. **b** Quantification of LDL in plasma from 4 weeks CD or HFD-fed mice, n=4 mice per diet group. **c** Body weight of mice before and after 4 weeks of HFD feeding, n=22 mice. **d** Quantification of blood glucose in PB, n=8 mice per diet group. Data show mean ± SD; ns, non-significant (*P*>0.05); ***, *P*<0.001 (two-tailed unpaired Student's *t*-test).

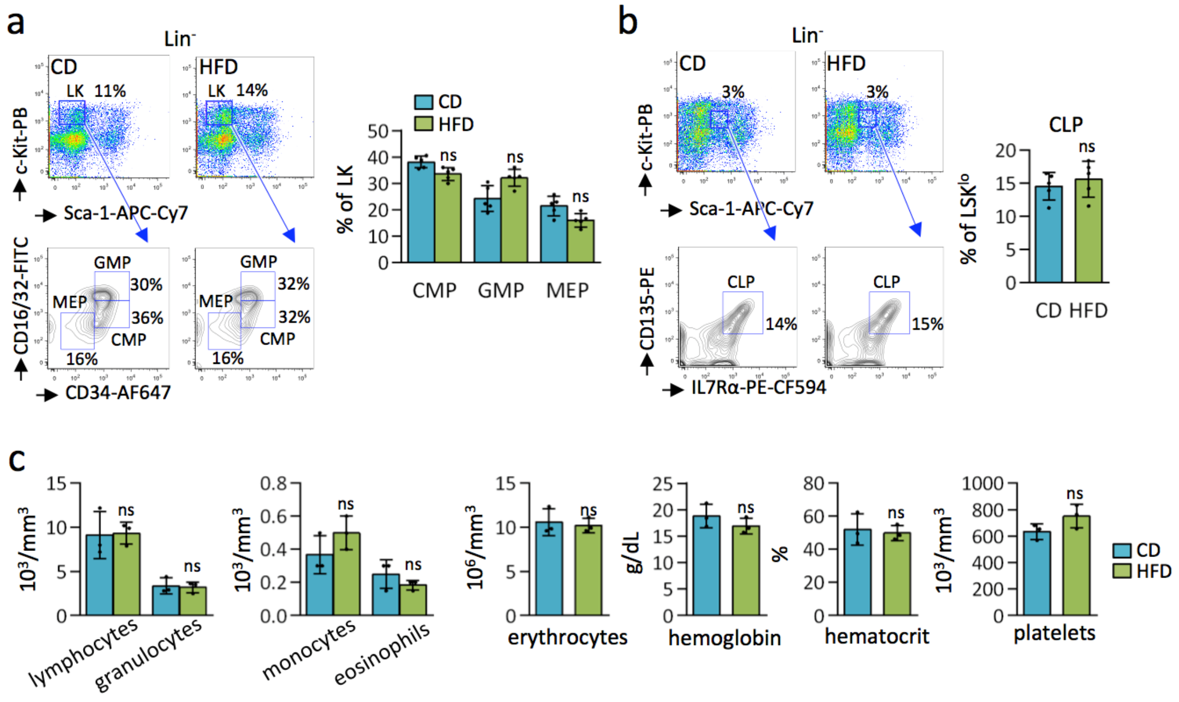

Supplementary Figure 2

Short-term HFD has no effect on progenitors in BM and PB constituents. **a** LK cells from mice fed a HFD for 4 weeks show no disturbance among progenitors. Granulocyte/Macrophage Progenitor (GMP), Mega-Erythroid Progenitor (MEP) and Common Myeloid Progenitor (CMP), n=5 mice per diet group. Gating strategy is shown on the left panel. **b** No disturbance is found among Common Lymphoid Progenitor (CLP), n=5 mice per diet group. Gating strategy is shown on the left panel. **c** Four weeks of a HFD have no impact on PB composition, n=3 mice per diet group. Data show mean ± SD; ns, non-significant (*P*>0.05) (two-tailed unpaired Student's *t*-test).

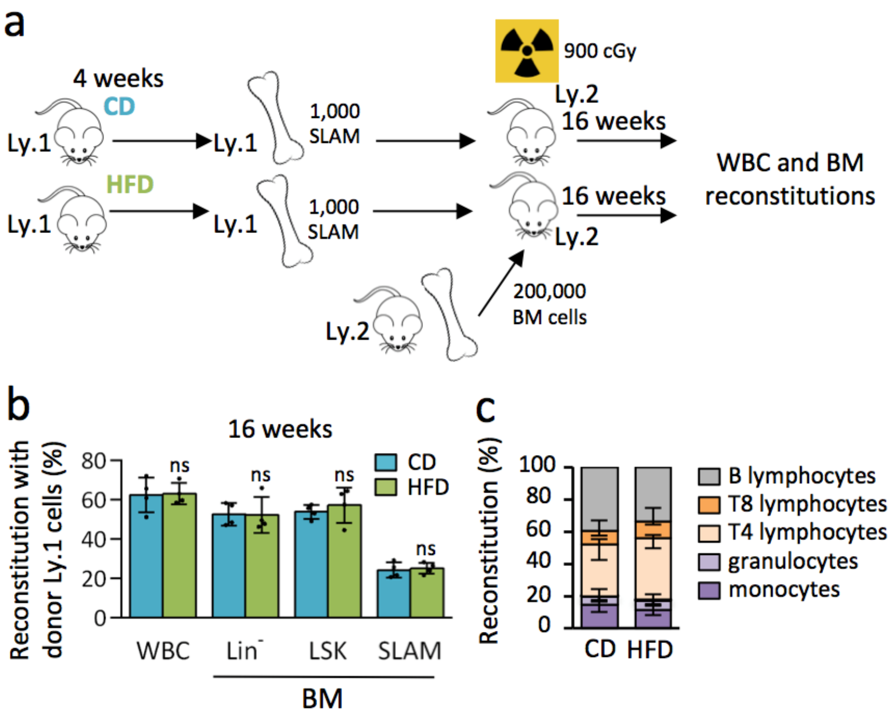

Supplementary Figure 3

Short-term HFD does not impact the ability of the remaining SLAM to reconstitute normal hematopoiesis following transplantation. **a** Experimental workflow. **b** Remaining SLAM from HFD-fed mice have the same capacity to reconstitute hematopoiesis function in BM and PB, 16 weeks after the transplantation, n=4 recipient mice per experimental condition; ns, non-significant (P>0.05) (two-tailed unpaired Student's t-test). **c** No difference is observed in PB reconstitution among different hematopoietic lineages, 16 weeks after the transplantation, n=4 mice per experimental condition. Data show mean ± SD.

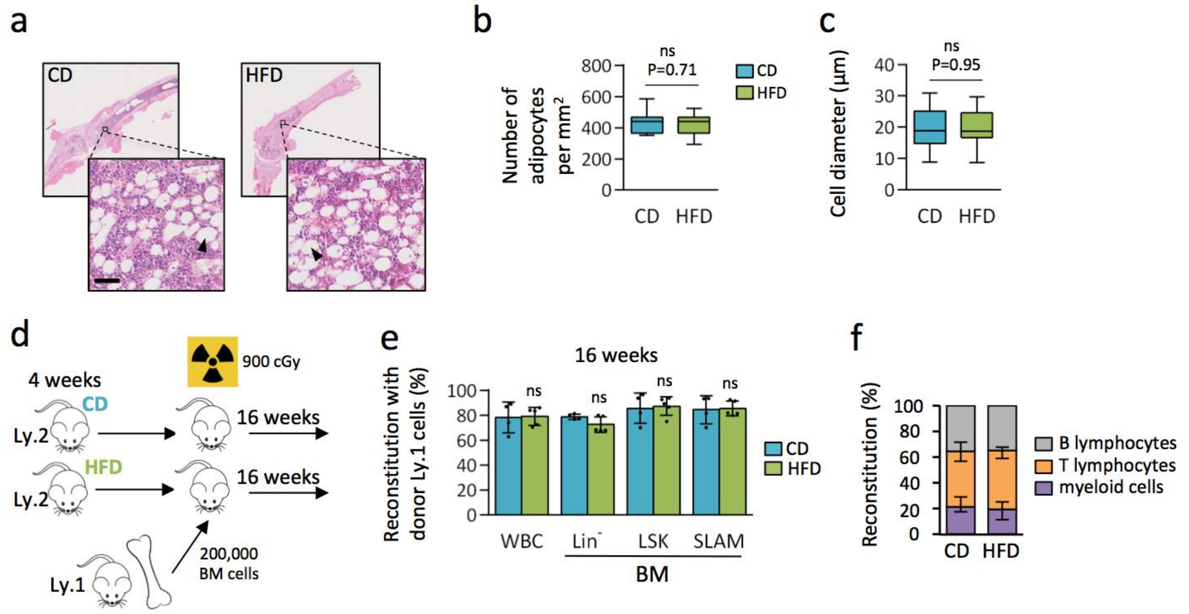

Supplementary Figure 4

Short-term HFD does not impact adipocyte cell content and size in BM, and the ability of the BM niche in hematopoietic reconstitution. **a** Digital images of paraffin-embedded, hematoxylin and eosin-stained BM for each diet group. Black arrowheads point to example of adipocyte. Black scale bar represents 50µm. **b** Adipocyte cell number per mm^2 and **c** size (n=50 cells per mouse for each diet) are determined from digital images, n=3 mice per diet group. **d** Experimental workflow. **e** HFD-fed mice have the same capacity than CD-fed mice to reconstitute hematopoiesis in BM and PB, 16 weeks after the transplantation of 200,000 total BM cells extracted from CD mice (mice per diet group: n=4 (CD) and n=5 (HFD)). **f** No difference is observed in PB reconstitution among different hematopoietic lineages, 16 weeks after the transplantation (mice per diet group: n=4 (CD) and n=5 (HFD)). T lymphocytes represent T4 and T8 lymphocytes. Myeloid cells represent monocytes and granulocytes. (b, c) Data are presented as median (central line), first and third quartiles (bottom and top of boxes, respectively), and whiskers (extreme values) (e, f) Data show mean ± SD; ns, non-significant (P>0.05) (two-tailed unpaired Student's t-test).

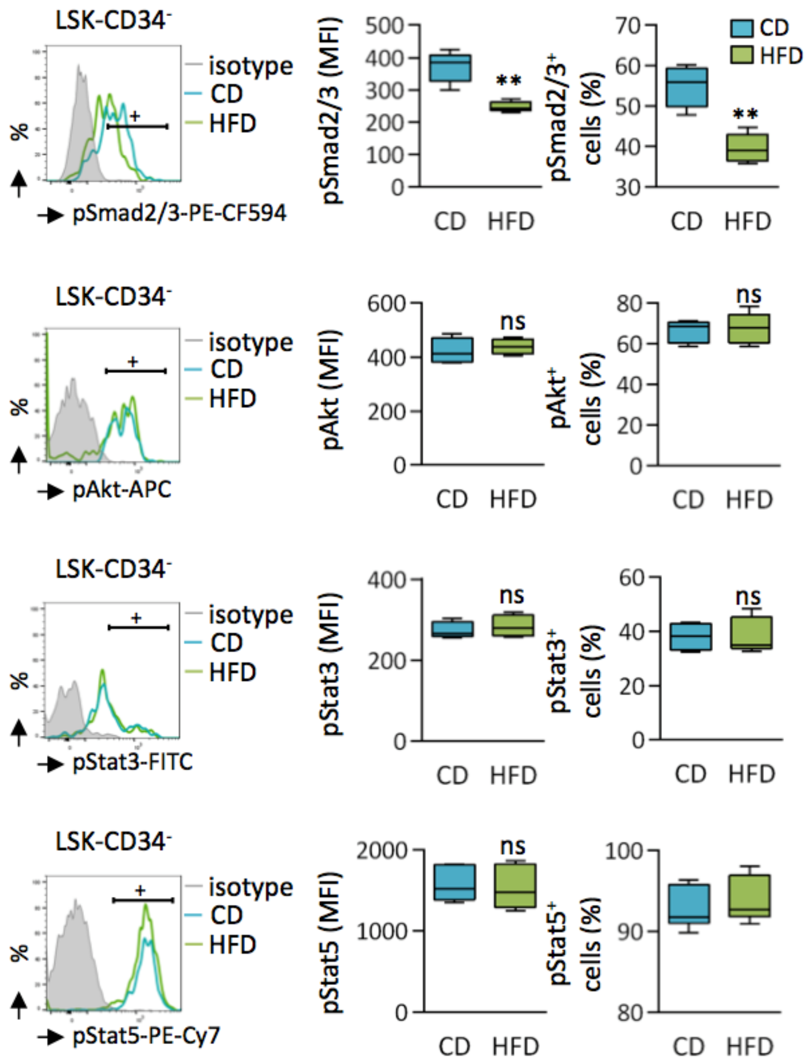

Supplementary Figure 5

Short-term HFD perturbs phosphorylation of Smad2/3 in LSK-CD34- HSC. The pSmad2/3, pAkt, pStat3 and pStat5 are detected by flow cytometry and results show their expression (MFI; median fluorescence intensity) and proportion (%) of positive (+) cells among LSK-CD34- HSC, n=5 mice per diet group. Data are presented as median (central line), first and third quartiles (bottom and top of boxes, respectively), and whiskers (extreme values); ns, non-significant (P>0.05); **, P<0.01 (two-tailed unpaired Student's t-test).

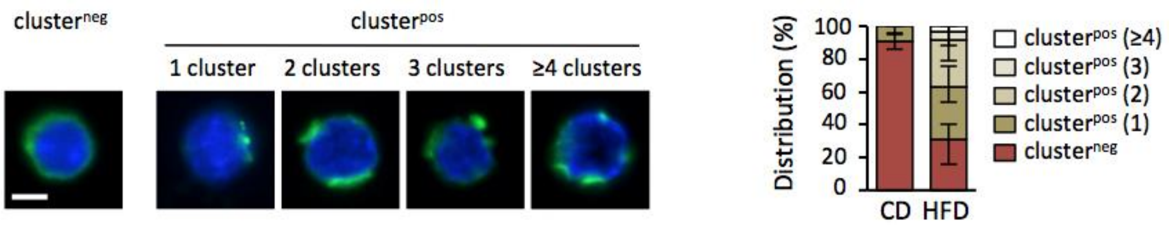

Supplementary Figure 6
Single-cell immunostaining on LSK-CD34$^-$ LR^{hi} cells showing that HFD induces formation of one, two, three or more LR clusters. Representative photographs of LSK-CD34$^-$ LR^{hi} HSC showing LR clusters (CTB, green) on the cell surface following a short term feeding with a HFD. Statistics of the proportion of cells (n>40 cells per mouse for each diet) for each condition is shown on the right panel, n=2 mice per diet group. Microscopy was performed on LSK-CD34$^-$ LR^{hi} cells isolated by FACS. White scale bar represents 5µm. Data show mean ± SD.

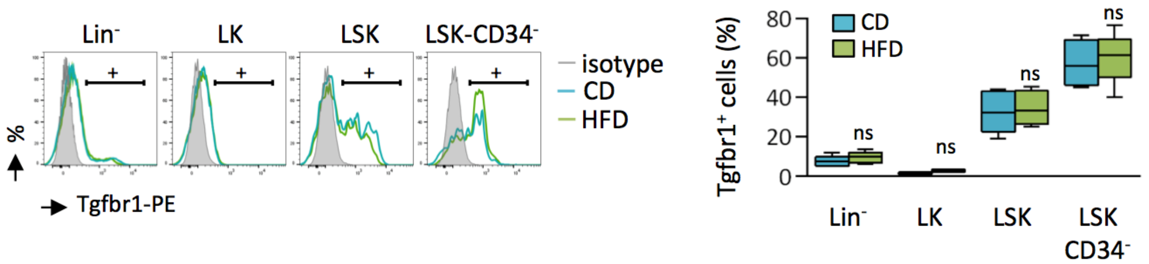

Supplementary Figure 7
Expression of the Tgfbr1 on HSC is not affected by HFD. The Tgfbr1 is detected by flow cytometry on different primitive hematopoietic cell populations and its expression is unchanged on the cell surface of LSK-CD34$^-$ cells following a short term feeding with a HFD. MFI; median fluorescence intensity. Data are presented as median (central line), first and third quartiles (bottom and top of boxes, respectively), and whiskers (extreme values); n=4 mice per diet group; ns, non-significant (P>0.05) (two-tailed unpaired Student's t-test).

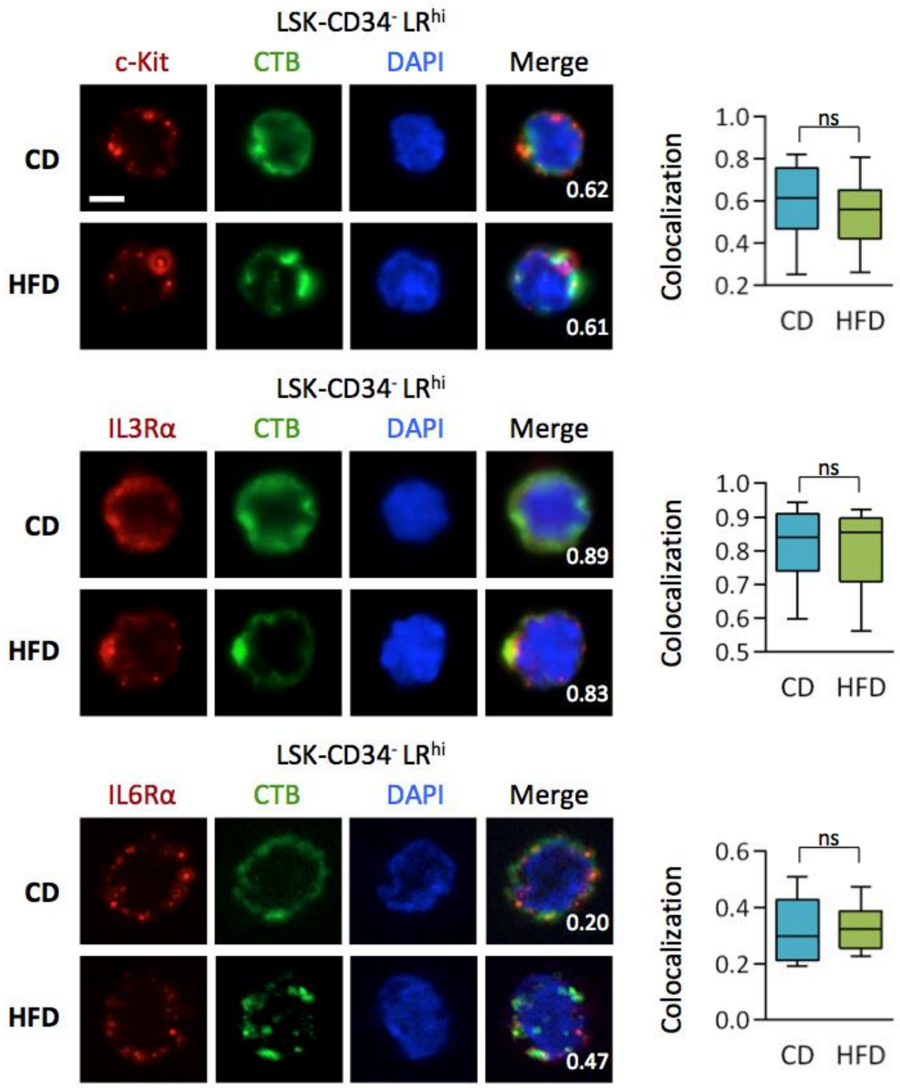

Supplementary Figure 8

Short-term HFD does not perturb the localization of c-Kit, IL3Rα and IL6Rα on the cell surface of LSK-CD34^{-} LR^{hi} cells. Data on the left panel shows examples of a representative single cell, with CTB (green) and cytokine receptor staining (red) for CD and HFD-fed mice. Microscopy was performed on LSK-CD34^{-} LR^{hi} cells isolated by FACS. White scale bar represents 5µm. Box-and-whisker plots showing statistics of the colocalization between CTB and specific cytokine receptor staining (n>40 cells per mouse for each diet) are shown on the right panel, n=2 mice per diet group. Data are presented as median (central line), first and third quartiles (bottom and top of boxes, respectively), and whiskers (extreme values); ns, non-significant (*P*>0.05) (two-tailed unpaired Student's *t*-test).

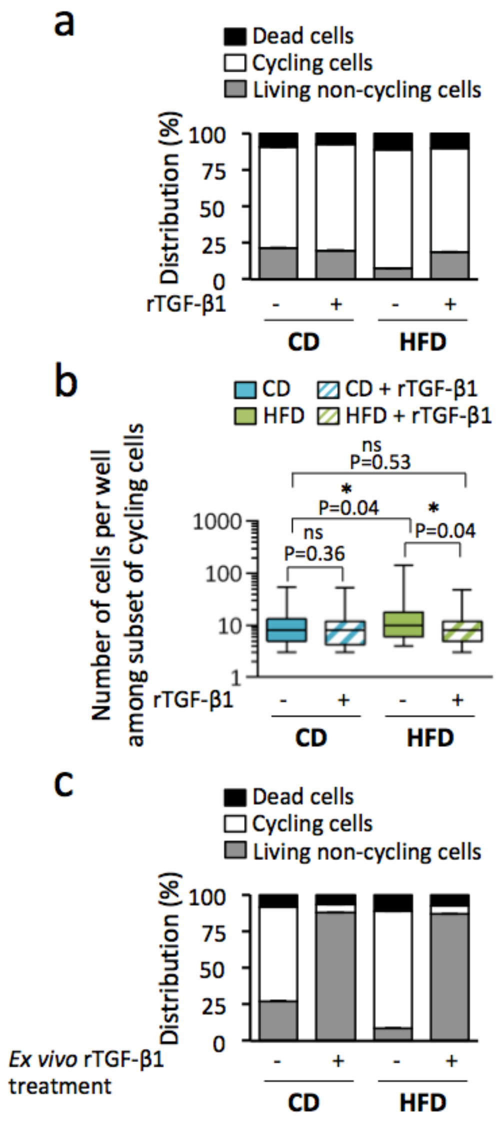

Supplementary Figure 9

Short-term HFD increases the proportion of cycling LSK-CD34$^-$ HSC cells, an effect which is compensated by injection of recombinant TGF-β1 in mice during the 4-week diet. **a** Freshly sorted single cell LSK CD34$^-$ HSC/well from CD or HFD-fed mice, treated or not with recombinant TGF-β1 (rTGF-β1 injected *in vivo* at 1.3µg/kg) twice per week during the 4 weeks of the diet, is cultured *ex vivo* for 5 days. After the culture period, cell viability and cell numbers are assessed (cells per mouse for each experimental condition: n=92 (CD), n=95 (CD + rTGF-β1), n=91 (HFD) and n=90 (HFD + rTGF-β1)). Bars show the proportion of dead, cycling, and living non-cycling cells for each condition. **b** Box-and-whisker plots showing the number of cells per well among colonies (colonies per mouse for each experimental condition: n=62 (CD), n=68 (CD + rTGF-β1), n=68 (HFD) and n=64 (HFD + rTGF-β1)) scored on day 5. Data are presented as median (central line), first and third quartiles (bottom and top of boxes, respectively), and whiskers (extreme values); ns, non-significant (P>0.05); *, P<0.05 (two-tailed unpaired Student's *t*-test). **c** Freshly sorted single cell LSK CD34$^-$ HSC/well from CD or HFD-fed mice is cultured *ex vivo* for 5 days in presence or absence of rTGF-β1 (10 ng/mL). After the culture period, cell viability and cell numbers are assessed (cells per mouse for each condition: n=73 (CD), n=72 (CD + rTGF-β1), n=68 (HFD) and n=81 (HFD + rTGF-β1)). Bars show the proportion of dead, cycling, and living non-cycling cells for each condition.

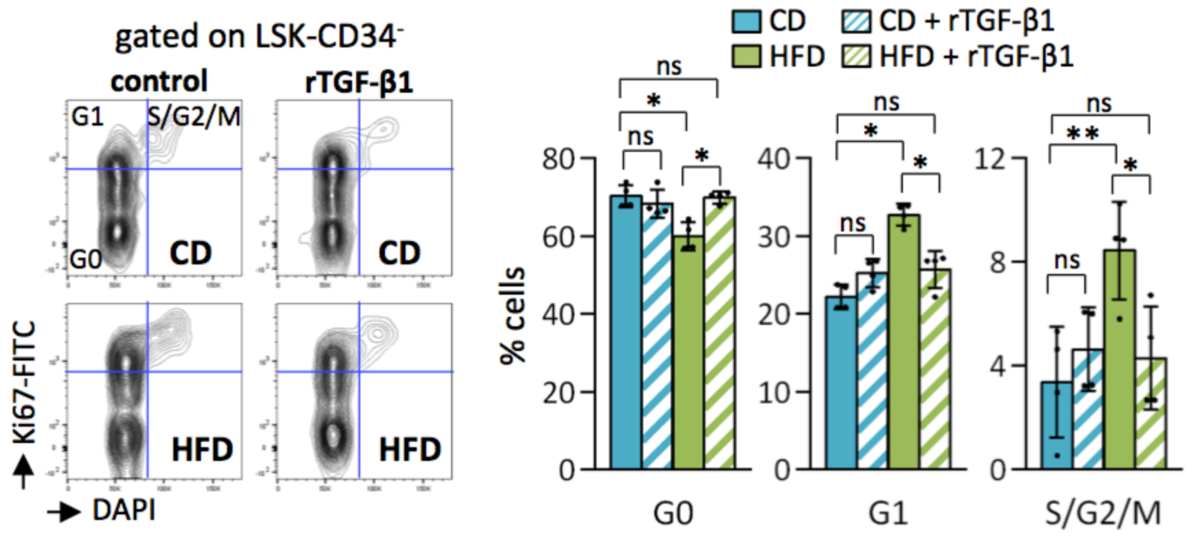

Supplementary Figure 10

Injection of recombinant TGF-β1 in mice compensates the HFD-mediated re-entry of HSC into the cell cycle. Recombinant TGF-β1 (rTGF-β1) is injected at 1.3 µg/kg twice per week into the tail vein of mice during the 4 weeks of the CD or HFD, n=4 mice per experimental condition. The Ki67 and DAPI staining is analyzed by flow cytometry on LSK-CD34$^-$ HSC. Data show mean ± SD; ns, non-significant (P>0.05); *, P<0.05; **, P<0.01 (two-tailed unpaired Student's *t*-test).

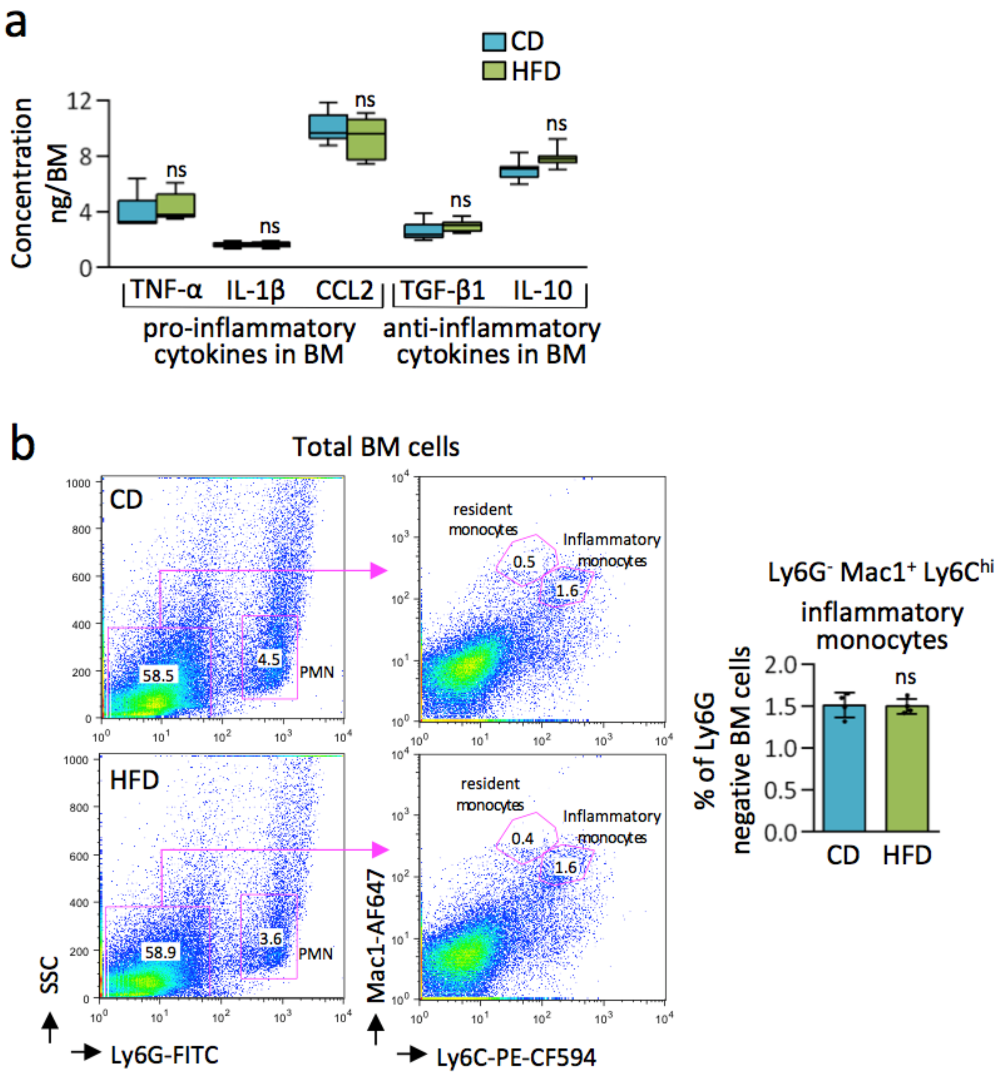

Supplementary Figure 11

Short-term HFD has no effect on inflammation in BM. **a** Quantification of pro- or anti-inflammatory cytokines in BM from CD or HFD-fed mice, n=4 mice per diet group. Data are presented as median (central line), first and third quartiles (bottom and top of boxes, respectively), and whiskers (extreme values). **b** Quantification of inflammatory monocyte levels in BM from CD or HFD-fed mice, n=4 mice per diet group. Gating strategy is shown on the left panel. Data show mean ± SD; ns, non-significant (P>0.05) (two-tailed unpaired Student's t-test).

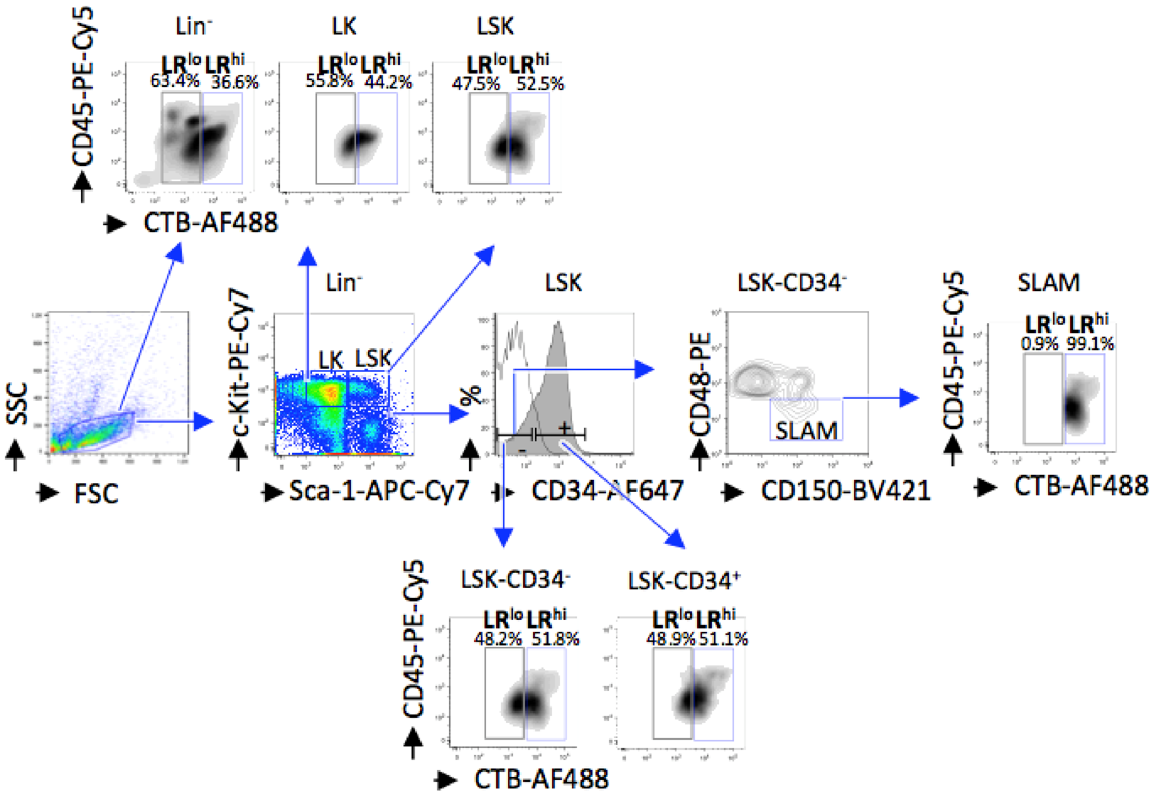

Supplementary Figure 12
Flow cytometry-gating strategy used for **Fig.1a**. The same gating strategy was used for **Fig.1c**.

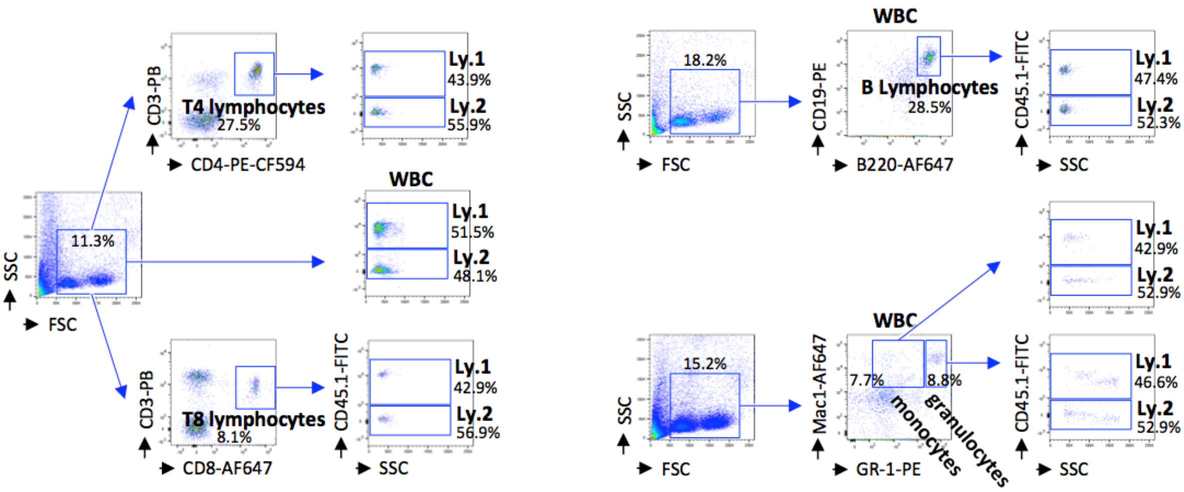

Supplementary Figure 13
Flow cytometry-gating strategy used for **Fig.7c-d.**

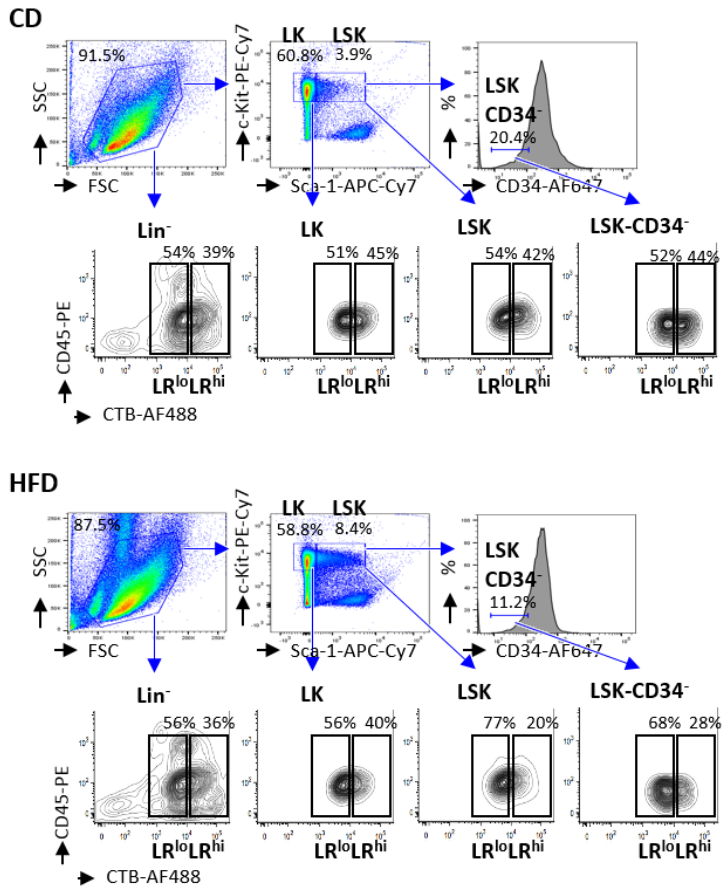

Supplementary Figure 14
Flow cytometry-gating strategy used for **Fig.2.** The same gating strategy was used for **Fig.1b**.

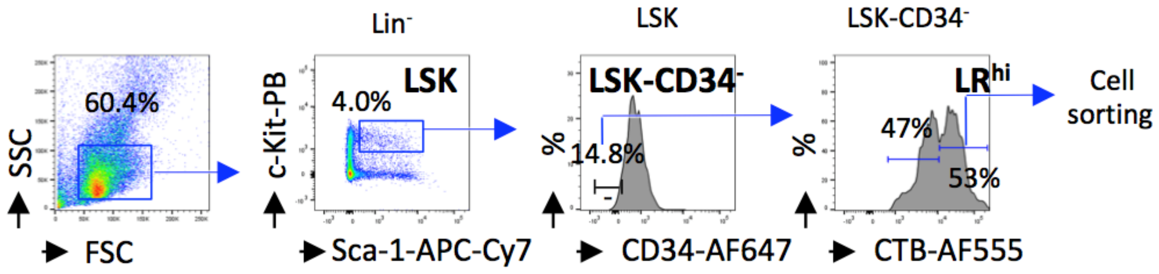

Supplementary Figure 15
Flow cytometry-gating strategy used for **Fig.6b**. The same gating strategy was used for **Fig.5a-d**, **Fig.6a-c**, **Fig.8**, **Fig.9** and **Supplementary Fig.5-10**. Different fluorochromes-conjugated antibodies or CTB were used following the same gating strategy.

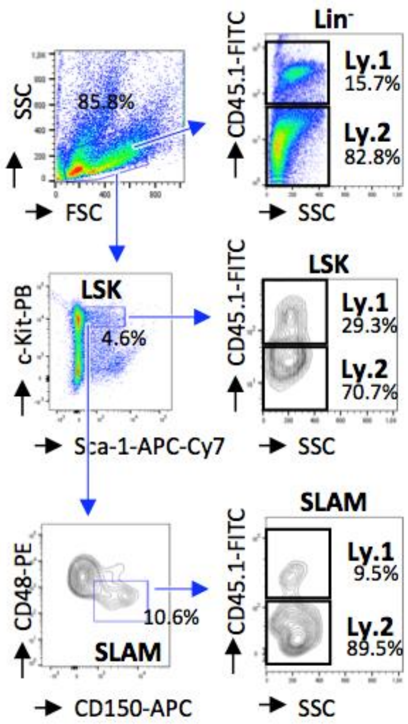

Supplementary Figure 16
Flow cytometry-gating strategy used for **Fig.4b.** The same gating strategy was used for **Fig.7a**, **c** and **Supplementary Fig.3b**, **4e**.

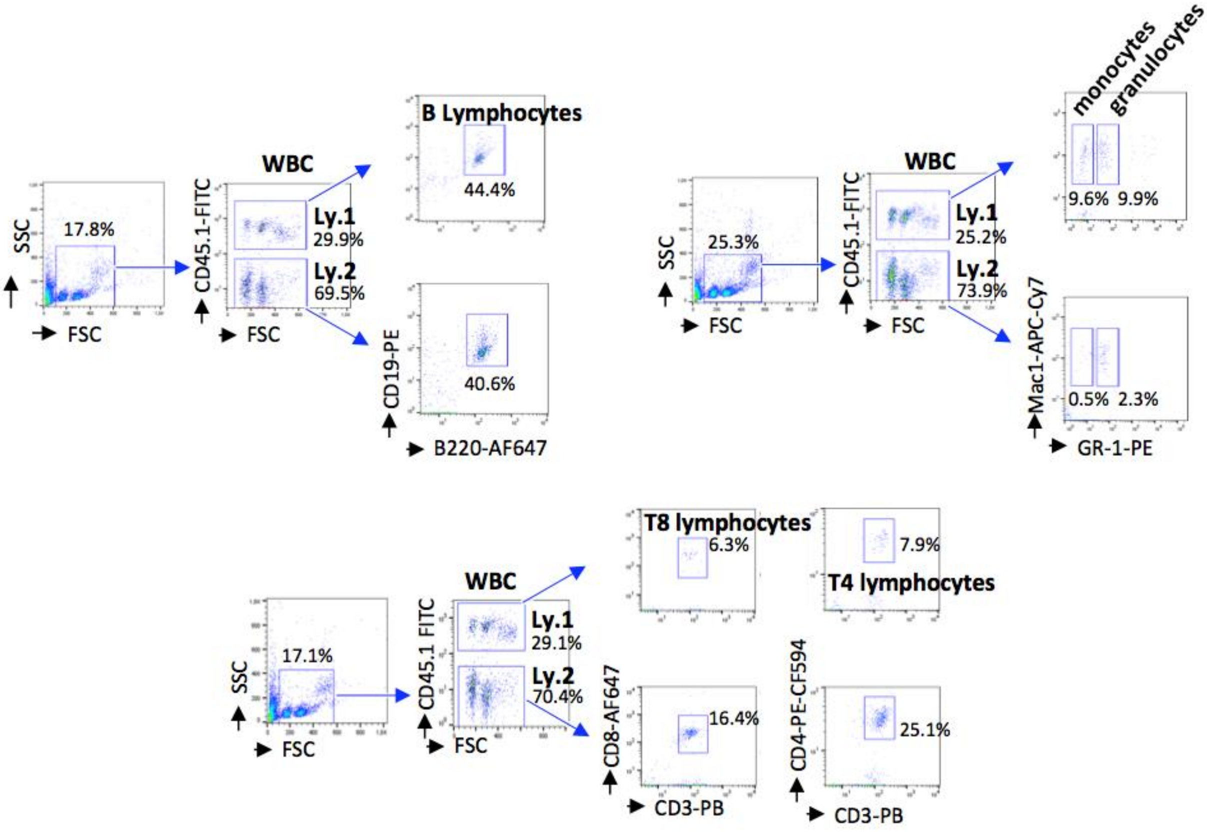

Supplementary Figure 17
Flow cytometry-gating strategy used for **Fig.4b-c**, **Fig.7d** and **Supplementary Fig.3b-c** and **4e-f**.

Supplementary Tables

Name	Common Name	Control feed pellets Mean	Control feed pellets Unit	Control feed pellets SD	High-fat feed pellets Mean	High-fat feed pellets Unit	High-fat feed pellets SD
**Fatty acid**		27239	µg/g	1385	178880	µg/g	10782
**Saturated fat**		7013		52	129244		9932
C8:0	Caprylic acid	3		2	61		41
C10:0	Capric acid	157		11	3252		629
C11:0	Undecylic acid	68		4	199		16
C12:0	Lauric acid	789		168	8837		641
C13:0	Tridecylic acid	39		6	271		12
C14:0	Myristic acid	327		33	24434		1428
C15:0	Pentadecylic acid	97		3	2811		136
C16:0	Palmitic acid	4405		130	69037		5653
C17:0	Margaric acid	59		0	1226		41
C18:0	Stearic acid	891		121	18514		1303
C19:0	Nonadecylic acid	10		0	107		6
C20:0	Arachidic acid	73		5	249		15
C21:0	Heneicosylic acid	7		0	47		5
C22:0	Behenic acid	18		1	32		1
C23:0	Tricosylic acid	5		0	15		1
C24:0	Lignoceric acid	23		1	30		0
C25:0	Pentacosylic acid	10		0	18		0
C26:0	Cerotic acid	33		2	105		5
**Monounsaturated fat**		6301		392	44626		131
C14:1 n-5	Myristoleic acid	2		0	2002		129
C16:1 n-7	Palmitoleic acid	241		0	3769		129
C17:1 n-8	Heptadecanoic acid	32		2	564		24
C18:1 n-9	Oleic/Elaidic acid (cis/trans)	5515		336	37903		105
C20:1 n-9	Gondoic acid	43		6	242		2
C20:1 n-7	Paullinic acid	365		35	110		4
C22:1	Erucic acid	53		8	18		1
C24:1 n-9	Nervonic acid	49		9	17		2
**Polyunsaturated fat**		13925		480	5010		413
C18:3 n-6	γ-Linolenic acid	5		2	59		6
C18:2 n-6	Linoleic acid	12150		439	3350		315
C18:3 n-3	α-Linolenic acid	1123		21	861		75
C20:4 n-6	Arachidonic acid	35		3	226		13
C20:3 n-9	Eicosatrienoic acid	0		0	24		2
C20:3 n-6	Dihomo-γ-linolenic acid	6		1	172		9
C20:5 n-3	Eicosapentaenoic acid (EPA)	211		11	105		7
C22:4 n-6	Docosatetraenoic acid	29		11	48		20
C22:5 n-3	Docosapentaenoic acid	50		2	149		5
C22:6 n-3	Docosahexaenoic acid (DHA)	318		13	15		1
**Cholesterol**		104	µg/g	0	2096	µg/g	0

Supplementary Table 1
Lipidomic assays performed by mass spectrometry on control feed pellets and high-fat feed pellets (MD. 88137, Envigo RMS Division, Indianapolis, IN).
