# Supplementary material for: High-fat diet disturbs lipid raft/TGF-β signaling-mediated maintenance of hematopoietic stem cells in mouse bone marrow
Source: Nat Commun. 2019 Jan 31;10:523. doi: 10.1038/s41467-018-08228-0 (PMC6355776; doi:10.1038/s41467-018-08228-0)
Supplement: Supplementary file 3 — Reporting Summary [file 41467_2018_8228_MOESM3_ESM.pdf]

nature research

Corresponding author(s): Ronan Quéré

Reporting Summary

Nature Research wishes to improve the reproducibility of the work that we publish. This form provides structure for consistency and transparency in reporting. For further information on Nature Research policies, see Authors & Referees and the Editorial Policy Checklist.

Statistical parameters

When statistical analyses are reported, confirm that the following items are present in the relevant location (e.g. figure legend, table legend, main text, or Methods section).

n/a	Confirmed	
☐	☒	The exact sample size (n) for each experimental group/condition, given as a discrete number and unit of measurement
☐	☒	An indication of whether measurements were taken from distinct samples or whether the same sample was measured repeatedly
☐	☒	The statistical test(s) used AND whether they are one- or two-sided<br>*Only common tests should be described solely by name; describe more complex techniques in the Methods section.*
☒	☐	A description of all covariates tested
☒	☐	A description of any assumptions or corrections, such as tests of normality and adjustment for multiple comparisons
☐	☒	A full description of the statistics including central tendency (e.g. means) or other basic estimates (e.g. regression coefficient) AND variation (e.g. standard deviation) or associated estimates of uncertainty (e.g. confidence intervals)
☐	☒	For null hypothesis testing, the test statistic (e.g. F, t, r) with confidence intervals, effect sizes, degrees of freedom and P value noted<br>*Give P values as exact values whenever suitable.*
☒	☐	For Bayesian analysis, information on the choice of priors and Markov chain Monte Carlo settings
☒	☐	For hierarchical and complex designs, identification of the appropriate level for tests and full reporting of outcomes
☒	☐	Estimates of effect sizes (e.g. Cohen's d, Pearson's r), indicating how they were calculated
☐	☒	Clearly defined error bars<br>*State explicitly what error bars represent (e.g. SD, SE, CI)*

Our web collection on statistics for biologists may be useful.

Software and code

Policy information about availability of computer code

Data collection	*Not Applicable*
Data analysis	Not Applicable

For manuscripts utilizing custom algorithms or software that are central to the research but not yet described in published literature, software must be made available to editors/reviewers upon request. We strongly encourage code deposition in a community repository (e.g. GitHub). See the Nature Research guidelines for submitting code & software for further information.

Data

Policy information about availability of data

All manuscripts must include a data availability statement. This statement should provide the following information, where applicable:

- Accession codes, unique identifiers, or web links for publicly available datasets
- A list of figures that have associated raw data
- A description of any restrictions on data availability

The data that support the findings of this study are available from the corresponding author upon reasonable request.

Field-specific reporting

Please select the best fit for your research. If you are not sure, read the appropriate sections before making your selection.

☒ Life sciences ☐ Behavioural & social sciences ☐ Ecological, evolutionary & environmental sciences

For a reference copy of the document with all sections, see nature.com/authors/policies/ReportingSummary-flat.pdf

Life sciences study design

All studies must disclose on these points even when the disclosure is negative.

Sample size	*For main experiments, at least 4 or 5 mice were used. No statistical methods were used to predetermine the sample size.*
Data exclusions	*Concerning bone marrow cell transplantation assays, some of the animals (<2% of transplanted mice) died between 15 and 30 days after transplantation likely due to aplasia . Dead mice were excluded from experimental groups.*
Replication	*The main findings of our work, such as HFD-mediated loss of HSC Lipid Raft (Figure 2 and Figure 6a) and HFD-mediated HSC attrition (Figure 3, Figure 7a) were reproduced at least two times. Moreover we demonstrated and confirmed that the HFD disturbs the Smad2/3-dependent TGF-β signaling thanks to several experimentations (localisation of Tgfbr1 within lipid rafts (Figure 6c, Figure 8 and Figure 9), analysis of phosphorylation of Smad2/3 by microscopy (Figure 8 and Figure 9a), and cytometry (Figure 5d, Figure 6a, Figure 9b and Supplementary Figure 5) analysis of quiescence thanks to KI67 staining (Figure 5a and Supplementary Figure 10).*
Randomization	*Animals were randomized to diet experimental groups.*
Blinding	*N/A*

Reporting for specific materials, systems and methods

Materials & experimental systems

n/a	Involved in the study	
☒	☐	Unique biological materials
☐	☒	Antibodies
☒	☐	Eukaryotic cell lines
☒	☐	Palaeontology
☐	☒	Animals and other organisms
☒	☐	Human research participants

Methods

n/a	Involved in the study	
☒	☐	ChIP-seq
☐	☒	Flow cytometry
☒	☐	MRI-based neuroimaging

Antibodies

Antibodies used

CD4-PE-CF594 (562285, dilution ratio 1:100), CD8-AF647APC (557682, dilution ratio 1:100), Mac1-APC-Cy7 (557657, dilution ratio 1:100), Mac1-PE-Cy7 (552850, dilution ratio 1:100), Mac1-AF647 (557686, dilution ratio 1:100), GR-1-FITC (553127, dilution ratio 1:100), GR-1-PE (553128, dilution ratio 1:100) antibodies (BD Biosciences) and CD19-PE (557399, dilution ratio 1:100), CD3-PB (558214, dilution ratio 1:100), B220-AF647 (103226, dilution ratio 1:100) antibodies (Biolegend). c-Kit-PE-Cy7 (558163, dilution ratio 1:100), CD34-AF647 (560230, dilution ratio 1:50), CD34-FITC (553733, dilution ratio 1:50), CD135-PE (553842, dilution ratio 1:100), CD48-PE (557485, dilution ratio 1:50), CD48-BV421 (562745, dilution ratio 1:50), CD45-PE-Cy5 (561870, dilution ratio 1:100), CD45-PE (553081, dilution ratio 1:100), CD16/32-FITC (553144, dilution ratio 1:100), IL7Rα-PE-CF594 (562419, dilution ratio 1:100), Mac1-AF647 (557686, dilution ratio 1:100), Ly6C-PE-CF594 (562728, dilution ratio 1:100) (BD Biosciences) and c-Kit-PB (105820, dilution ratio 1:100), CD150-APC (115910, dilution ratio 1:100), CD150-BV421 (115925, dilution ratio 1:100), Sca-1-APC-Cy7 (108126, dilution ratio 1:100), Ly6G-FITC (127605, dilution ratio 1:100) (Biolegend). To separate donor cells from support and recipient cells, CD45.1 (Ly.1)-FITC (553775, BD Biosciences, dilution ratio 1:100) antibody was used (BD Biosciences). AF488- (C-34775, 1 µg/mL) or AF555- (C-34776, 1 µg/mL) conjugated cholera toxin subunit B (Thermo Fisher Scientific) were used to stain LR. For studies on cell cycle and quiescence, Ki67-FITC antibody (556026, BD Biosciences, dilution ratio 1:6) was used (BD Biosciences). Tgfbr1 was stained with anti-Tgfbr1-PE antibody (FAB5871P, R&D Systems, dilution ratio 1:100). For intracellular protein staining, anti-phospho-Smad2(S465/S467)/Smad3(S423/S425)-PE-CF594 (562697, BD Biosciences, dilution ratio 1:20), anti-phospho-Stat5(Y694)-PE-Cy7 (560117, BD Biosciences, dilution ratio 1:6), anti-phospho-Akt(S473)-APC (130-105-293, Miltenyi Biotec, dilution ratio 1:11) and anti-phospho-Stat3(Y705)-FITC (651019, Biolegend, dilution ratio 1:20) antibodies (BD Biosciences). For colocalization of LR and Tgfbr1, we used AF555-conjugated CTB (C-34776, Thermo Fisher Scientific, 1 µg/mL) and anti-Tgfbr1 (PA5-38718, Thermo Fisher Scientific, dilution ratio 1:100) with secondary anti-rabbit AF488 (A27034, Thermo Fisher Scientific, dilution ratio 1:1000) antibodies. For the phospho-Smad2/3 microscopy, cells were

stained with the cell surface markers (LSK-CD34-) and AF555-conjugated CTB (C-34776, Thermo Fisher Scientific, 1 µg/mL) and with an anti-phospho-Smad2(S465/S467) antibody (clone AB3849, Merck, dilution ratio 1:250) and with secondary anti-rabbit AF488 (A27034, Thermo Fisher Scientific, dilution ratio 1:1000) antibody.
For the study of several receptors on the cell surface, LR were stained with AF488-conjugated CTB (C34775, Thermo Fisher Scientific, 1 µg/mL) and we used biotin-conjugated c-Kit/CD117 (553353, BD Pharmingen, dilution ratio 1:50), IL3Rα (106003, Biolegend, dilution ratio 1:50), IL6Rα (115803, Biolegend, dilution ratio 1:50) antibodies and Streptavidin-AF555 (S32355, Thermo Fisher Scientific, dilution ratio 1:500)

Validation

All the antibodies that we used are commercial antibodies. Validation statements are provided on the manufacture's website.

Animals and other organisms

Policy information about studies involving animals; ARRIVE guidelines recommended for reporting animal research

Laboratory animals

C57BL/6J (Ly.2) and congenic B6.SJL (Ly.1) mice were provided by Envigo, kept in the Animal Facility at the University of Burgundy, and used at age of 8-12 weeks for feeding or bone marrow transplantation.

Wild animals

N/A

Field-collected samples

N/A

Flow Cytometry

Plots

Confirm that:

☒ The axis labels state the marker and fluorochrome used (e.g. CD4-FITC).

☒ The axis scales are clearly visible. Include numbers along axes only for bottom left plot of group (a 'group' is an analysis of identical markers).

☒ All plots are contour plots with outliers or pseudocolor plots.

☒ A numerical value for number of cells or percentage (with statistics) is provided.

Methodology

Sample preparation

White blood cell analyses:
Peripheral blood was collected from the tail vein, and erythrocytes were lysed with a hemolytic buffer (150nM NH4Cl, 10mM KHCO3, 0.1mM ethylene-diamine-tetra-acetic acid). The remaining cells were stained with conjugated antibodies for flow cytometry analysis.
Bone marrow analyses:
Hind limb bones were crushed in a mortar and total BM cells were filtrated (30µm). Magnetically lineage-depleted BM cells (Lineage Cell Detection Cocktail-Biotin, 130-092-613, Miltenyi Biotec) were stained in phosphate-buffered saline (PBS), pH7.2, with combinations of antibodies for flow cytometry.

Instrument

Cell subsets in bone marrow and peripheral blood were analyzed on a FACS Canto10 flow cytometer (BD Biosciences). LSK-CD34- cells were sorted on a FACS Aria cell sorter (BD Biosciences).

Software

Data were collected and analyzed using BD FACSDiva v8.0.1 (BD Biosciences) and FlowJo software (TreeStar Inc), respectively.

Cell population abundance

We provided FACS-gating strategies for all flow cytometry analysis and FACS cell sorting that confirm the abundance of the analyzed or sorted relevant cell populations.

Gating strategy

We provided FACS-gating strategies for all flow cytometry analysis and FACS cell sorting, specifying the preliminary FSC/SSC gates and how positive and negative staining cell populations are defined.

☒ Tick this box to confirm that a figure exemplifying the gating strategy is provided in the Supplementary Information.
